# Identifying Optimal Wearable Devices for Monitoring Mobility in Hospitalized Older Adults: Feasibility, Acceptability, and Validity Study

**DOI:** 10.2196/64372

**Published:** 2025-05-12

**Authors:** Paulo Nascimento, Renata Kirkwood, Lauren E Griffith, Mylinh Duong, Cody Cooper, Yujiao Hao, Rong Zheng, Samir Raza, Marla Beauchamp

**Affiliations:** 1 School of Rehabilitation Sciences, McMaster University, 1280 Main Street West, Hamilton, ON, L8S 4L6, Canada, 1 (905) 525-9140; 2 Department of Health Research Methods, Evidence, and Impact, McMaster University, Hamilton, ON, Canada; 3 Population Health Research Institute, McMaster University, Hamilton, ON, Canada; 4 Department of Medicine, McMaster University, Hamilton, ON, Canada; 5 Department of Computing and Software, McMaster University, Hamilton, ON, Canada

**Keywords:** older adults, gerontology, geriatric, aging, feasibility, acceptability, mobility, wearable, inpatient, hospital-acquired disability, physical performance, mHealth, mobile health, hospital, physical activity, exercise, Fitbit, posture, walk

## Abstract

**Background:**

Hospitalized, frail older adults have an increased risk of developing hospital-acquired disability associated with hospital practices of restricted physical activity and immobilization. The use of activity tracking wearable devices may allow identification and prevention of mobility decline, reducing hospital-acquired disability.

**Objective:**

This study aimed to identify the optimal wearable device and wear location for monitoring mobility in older hospitalized patients. Specific objectives included (1) comparison of the feasibility and acceptability of ActiGraph wGT3X-BT (ActiGraph LLC), MOX1 (Maastricht Instruments), MetaMotionC (mBientLab), and Fitbit Versa (Google) for continuous mobility monitoring and (2) determination of the concurrent validity of the selected device for detecting body posture and step count.

**Methods:**

Participants were recruited for this observational study in the acute medical care unit of an academic hospital in Hamilton, Ontario, Canada. Eligible patients were aged 60 years and older, able to undertake the mobility protocol, and had an anticipated length of stay greater than 4 days. The study was divided into 2 experiments. Experiment 1 evaluated the feasibility of 4 wearable devices and validated the derived data for body posture and step count. Experiment 2 involved a mobility assessment session and a 24-hour monitoring and feasibility period with the selected device from experiment 1.

**Results:**

The ActiGraph wGT3X-BT emerged as the most feasible device, demonstrating superior usability, data acquisition, and management. The thigh-worn ActiGraph accurately detected sedentary behavior, while the ankle-worn device provided detailed information on step counts and body postures. Bland-Altman plots and intraclass correlation coefficients indicated that the ankle-worn ActiGraph showed excellent reliability for step counting, with minimal bias and narrow limits of agreement. Patients expressed a high willingness to wear a continuous mobility tracking device at the hospital and at home.

**Conclusions:**

Thigh- and ankle-worn ActiGraph are optimal for assessing and monitoring mobility in older hospitalized patients. Challenges such as discomfort and device removal observed during the 24-hour monitoring period highlight areas for future studies. Overall, our findings support the integration of wearable technology in hospital settings to enhance mobility monitoring and early intervention strategies. Further research is warranted to evaluate the long-term use of wearable data for predicting health outcomes post hospitalization and informing clinical decision-making to promote early mobility.

## Introduction

Research indicates that a significant proportion of the adverse functional and health outcomes experienced by older adults during hospitalization may not be directly linked to their underlying health conditions or the reasons for hospitalization [1-3]. Instead, they may arise from certain hospital practices, such as restricted physical activity and immobilization, which may be harmful for older patients, especially for those who are frail [1]. Studies have consistently shown that older patients spend a minimal amount of time standing or walking during their hospital stay, typically just 3% of the time [4-7]. This limited mobility can have significant consequences for function, with each day spent in bed associated with a 1%‐5% loss in muscle strength [3]. On the other hand, there is evidence showing that older adults who regain their prehospitalization level of function after discharge have lower mortality rates and maintain their functional levels 1 year post discharge. Therefore, early detection and prevention of mobility decline during hospitalization is critical to improving patient outcomes and reducing health care utilization [8].

Wearable technology provides a direct means of assessing and monitoring mobility, by gathering continuous information on patients’ physical activities and mobility patterns. This capability would allow practitioners to create and monitor tailored mobility care for each patient, which may improve functional outcomes. Several wearable monitors have been validated in healthy individuals including among older adults living in the community [9,10]. However, experiment protocols, including population type, settings, sensor type, activity, and wear location (eg, wrist vs thigh) can influence the validity of these devices [11-14]. Despite the increased use of wearables in clinical settings, the feasibility, validity, and reliability of these devices have not been fully established in older hospitalized patients [15,16]. Collecting data accurately with wearables in hospitalized patients presents significant challenges, as they tend to be more sedentary and walk slower [17]. In addition, hospitalized patients often have medical devices attached to them, such as intravenous lines or heart monitors, which can impede mobility and make it challenging to select the optimal wear location for data collection.

The overall objective of this study was to identify the optimal wearable device and wear location for assessing and monitoring mobility in older hospitalized patients. Specifically, we aimed to (1) compare the feasibility and acceptability of different wearable devices to assess mobility for long-term continuous monitoring during inpatient hospital stays among older hospitalized patients, and (2) for the selected device from aim 1, determine its concurrent validity for detecting body posture and step count. To fulfill the study aims we performed 2 experiments with 2 independent samples. In the first experiment, we used a standardized protocol to test the feasibility of data collection with the ActiGraph wGT3X-BT, MOX 1, MetaMotionC (MMC), and Fitbit Versa over a short period of time, while validating the data collected for detecting body posture and step count using a standardized protocol. The second experiment was divided in 2 parts where patients wore the selected wearable from experiment 1 in 3 different body locations and performed a standardized activity protocol, followed by a 24-hour free-living protocol. This investigation was crucial for understanding the practicality of wearable devices for mobility monitoring in older adults in a hospital setting, as well as identifying potential barriers or limitations that may impact their utility.

## Methods

### Participants

Patients were recruited from the acute medical care unit of the Juravinski Hospital, Hamilton, Ontario, Canada. Inpatients were initially identified by the admitting physician, who sought approval before study personnel approached them. Patients were only included if, based on the judgment of the admitting physician, they were deemed capable of providing informed consent. A study coordinator, not directly involved in patient care, then contacted the eligible patients to obtain informed consent. During data collection, a hospital physical therapist, who accompanied all participants and assisted with data collection, ensured safety and facilitated communication when necessary.

Eligible patients were aged 60 years and older, able to undertake the mobility protocol with or without assistance, had an anticipated length of stay of more than 4 days, and able to provide written informed consent. Experiment 1 was conducted from November to December of 2019 and experiment 2 from September to December 2020.

### Ethical Considerations

This study was approved by the Hamilton Integrated Research Ethics Board (HiREB #7145) and all participants provided written informed consent before participation. At the time of consent, we recorded information regarding participants’ demographics, preadmission functional performance, and health status. All participants had the right to withdraw from the study at any time without any adverse consequences. All data were anonymized. Participants did not receive compensation for their participation.

### Measurement Instruments

For the feasibility and device performance of experiment 1, 4 devices were compared, that is, the MetaMotionC (MMC), Fitbit Versa 1, MOX1, and the medical-grade monitor ActiGraph wGT3X-BT. The MMC by mBientLab was chosen for its open platform enabling on-board programming, in addition to its lower cost. The Fitbit Versa 1 (Google) was widely recognized in the community at the study’s time, despite lacking direct access to raw sensor data extraction. For both devices, a Python algorithm was developed to extract the raw data. The MOX1 device (Maastricht Instruments , Netherlands) presents a triaxial accelerometer sensor and is equipped with proprietary software. During the study period, the proprietary software of the MOX1 did not offer direct access to the raw data and step count data. To address this limitation, the company provided us with a MATLAB function for extracting the raw data. Finally, the medical-grade ActiGraph wGT3X-BT (ActiGraph), often viewed as the gold standard in accelerometry movement analysis for research, features a built-in triaxial accelerometer that captures high-resolution raw acceleration data. The ActiLife software (ActiGraph LLC, version 6.11.4) was employed to initialize, process, and download data, extracting step counts and body posture measures (time spent lying down, sitting, and standing). In experiment 2, we used the ActiGraph wGT3X-BT as it was the selected device from experiment 1.

### Protocols

#### Experiment 1

Patients from the first experiment were engaged in the activity data procedures to test the feasibility of 4 different wearable devices (ActiGraph wGT3X-BT, MOX1, MMC, and Fitbit Versa), as well as the concurrent validity of the ActiGraph in detecting body posture and step counts. Each patient wore all 4 devices on the waist, 3 devices on the thigh, and 3 on the ankle simultaneously and interchangeably, that is, up to 10 devices per patient. An elastic band equipped with Velcro on both ends was used to attach the devices securely to the body. The waistband featured 4 pockets, while the thigh and ankle bands had 3 pockets each, providing the flexibility to interchange the devices as needed during the study. The waistband was positioned at the level of the anterior superior iliac spine, the thigh band above the kneecap, and the ankle band above the malleolus. After positioning the elastic bands, the wearable devices were randomly assigned to each pocket. Once wearable devices were placed, patients performed the mobility protocol that included body posture tasks (standing, sitting, and lying) and the timed up and go (TUG) [18] mobility test.

During the body posture tasks, patients were asked to lie down, sit on the edge of the bed, and then stand with or without support for 5 minutes. The time spent in each body posture was observed and recorded by a physiotherapist. Following, the patients performed the TUG which is a reliable and valid test to assess mobility and balance in older adults [18]. It measures, in seconds, the time taken by an individual to stand up from a standard armchair, walk 3 meters, turn, walk back to the chair, and sit down. The participants wore their customary walking aid (none, cane, or walker), and no physical assistance was given. The recording initiated as the patient raised from the chair and stopped as the patient sat on the chair again. The time and the number of steps taken to complete the test were also counted and recorded. Following the activity procedures, patients were invited to complete the acceptability questionnaire (Textbox 1). The protocol took approximately 45‐60 minutes to complete.

Textbox 1. **Feasibility questionnaires for experiments 1 and 2.**

**Experiment 1**

1. Have you ever used a device to measure physical activity in the past? (Responses: yes, no)
2. Would you be willing to wear the device for a longer period, 5 to 7 days, as part of a research study? (Responses: very likely, somewhat likely, not likely)
3. What part of the body would you prefer to wear the device? (Responses: waist, thigh, ankle)
4. Which of these devices would you likely use? (Responses: ActiGraph, MOX1, Versa, MetaMotionC)
5. How easy would it be for you to remember to use the device every day? (Responses: very easy, easy, very difficult, difficult)
6. Do you think this device would interfere with your daily routine? (Responses: no effect, minor effect, major effect)
7. Would you feel more motivated to move when wearing the device? (Responses: yes, no, no answer)

**Experiment 2**

1. Have you ever used a device to measure physical activity in the past? (Responses: yes, no)
  - What devices did you use?
  - What activity (or activities) did you track with the devices?
  - How long ago was it that you used the devices?
  - If you stopped using the devices, why did you stop?
2. Would you be willing to wear a device to measure physical activity for 5 to 7 days while in the hospital? (Responses: likely, uncertain, unlikely, very likely, very unlikely)
3. Would you be willing to wear a device daily to measure physical activity once you return home from the hospital, for a period of up to say 3 months? (Responses: yes, no)
4. What part of the body would you most prefer to wear the device? (Responses: wrist, waist, thigh, ankle, other)
5. Would you feel more motivated to move when wearing a device to measure physical activity? (Responses: yes, no)
6. Prior to being in the hospital, how many days per week on average did you engage in 30 minutes or more of physical activity, which was enough to raise your breathing rate? (Responses: 1, 2, 3, 4, 5, 6, 7, none)

#### Experiment 2

We conducted 1 mobility assessment session where patients wore the chosen device from experiment 1 (ActiGraph) in 3 different body locations (wrist, thigh, and ankle) and performed a standardized mobility protocol under the supervision of a trained physiotherapist. Patients wore the ActiGraph on the wrist using a wristband. At the thigh, the ActiGraph was attached to the anterior aspect of either the left or right thigh just above the kneecap, and at the ankle, the device was attached just above the malleoli. The Hypafix Stretch Non-Woven Adhesive (BSN Medical) was used to affix the devices. The mobility protocol included the same body posture protocol as the first experiment, followed by a step count task recorded during a 30-meter walking test (30MWT) [19,20]. Patients were asked to stand up from a chair, walk 30 meter, turn around, and walk back at a comfortable pace using their usual walking aid if necessary. The time and the number of steps taken to complete the test was counted and recorded. The mobility protocol in experiment 2 lasted approximately 30‐45 minutes.

**Figure 1.**
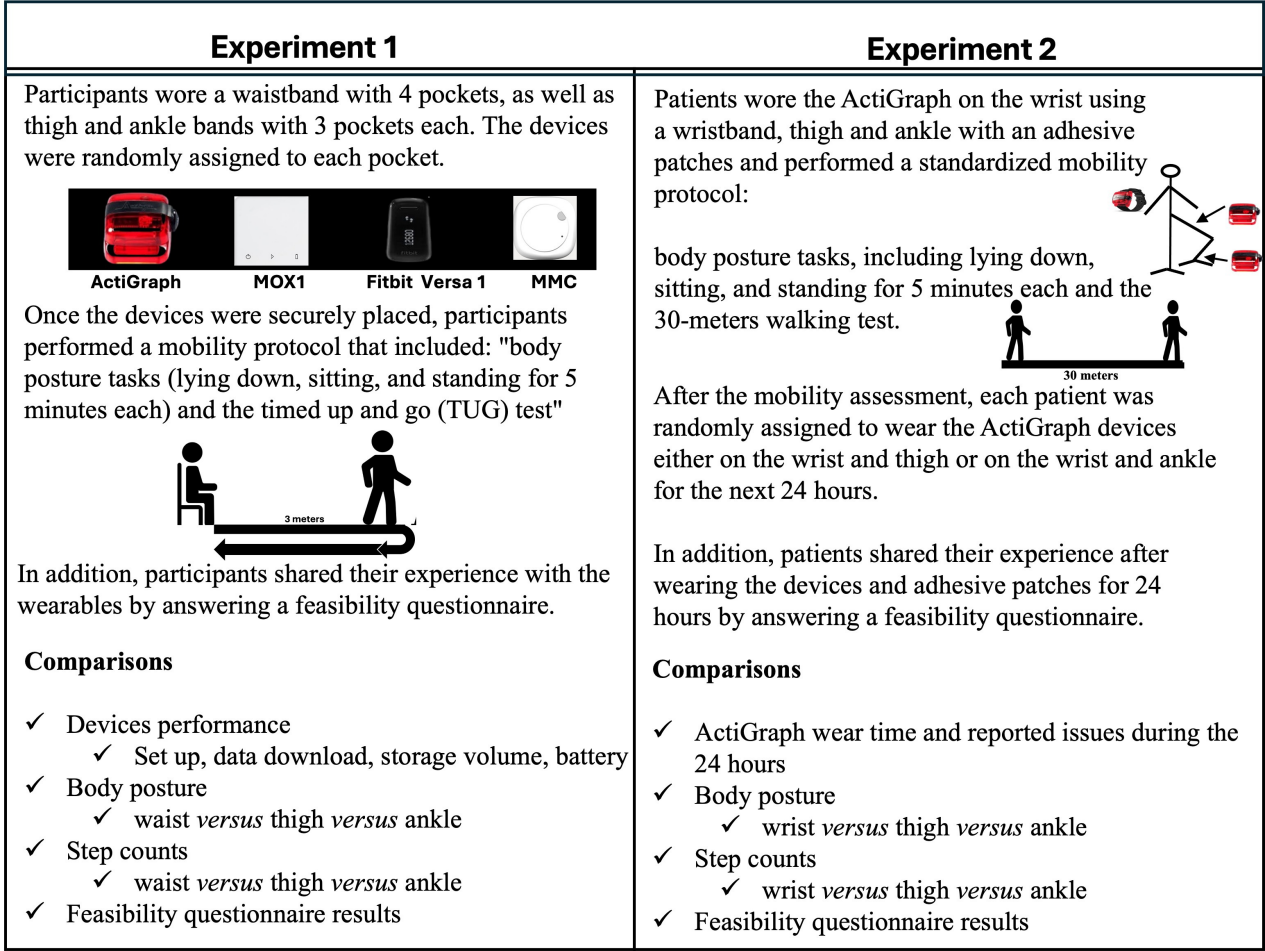
Summary of procedures for experiment 1 and 2.

After the mobility assessment, each patient was randomly assigned to wear the ActiGraph devices on either the wrist and thigh or wrist and ankle for the next 24 hours. We assessed the acceptability and feasibility of the wearable devices by location, as well as their acceptability of

wearing the adhesive patches over a 24-hour duration in the hospital. We also documented any interruptions or issues encountered during the 24-hour monitoring period. If patients removed or stopped using the device, the research assistant recorded feedback from both the patient and the attending nurses regarding the reasons for discontinuation. Figure 1 illustrated the variations within each experiment.

### Data Reduction

#### Experiment 1

Data were collected at 100 Hz for MOX1, and at 50 Hz for the other devices. In the second experiment, the ActiGraph data were collected at 30 Hz. Custom Python algorithms were created for the MMC and Fitbit devices to enable continuous saving and downloading of raw accelerometer data, necessitating the use of a companion device, a tablet. The MOX1 device was initialized using its proprietary software and data were downloaded using the provided MATLAB function. Raw data from these devices were not subjected to further processing; our focus was solely on recording downloading time, as well as the quantity and quality of the saved data.

In both experiments, the ActiLife software (version 6.11.4) was used to initialize, download, and process the ActiGraph data. Data were aggregated into 60 seconds time-stamped epochs. The following measures were obtained from the ActiLife: wear time, counts per minutes, activity intensity, step counts, and body posture (time spent in lying down, sitting, and standing). The following measures were obtained from the ActiLife algorithms: wear time, counts per minute, activity intensity, step counts, and body posture (time spent lying down, sitting, and standing). For step counts, since the ActiLife algorithms measures strides when attached to the thigh, we doubled the stride numbers to determine the actual number of steps taken. This adjustment is necessary because each stride recorded by the device corresponds to two steps—one for each leg—so the raw stride count is multiplied by 2 to obtain an accurate step count.

In addition, we applied the ActiGraph manufacturer’s step algorithm, the low-frequency extension filter, to the step count data. This filter is designed to detect lower-amplitude movements, enhancing the accuracy of step detection [21]. Body posture classification in both experiments was obtained using the thigh-worn algorithm from the ActiGraph that relies on movement and the thigh angle to accurately classify lying and sitting versus standing positions [22]. The first and last 45 seconds of data from each activity were discarded to avoid potential participant error in recording time and to exclude the transition times.

#### Experiment 2

For the 24-hour protocol, the accelerometer data from the ActiGraph was screened for wear time using the method described by Choi et al [23]. Based on the activity counts determined by the ActiLife algorithms, and using an epoch length of 60 seconds, nonwear time was defined as 90 consecutive minutes of zero counts, with an allowance of 2 minutes of nonzero counts, provided there were 30-minute consecutive zero counts before and after that allowance. Based on the wear time, we determined the average amount of time the patients wore the devices at the wrist, thigh, and ankle during the 24-hour protocol.

### Statistical Analysis

Descriptive data were analyzed using measures of central tendency and dispersion. Absolute percentage errors were calculated between the observed body posture time and step count against the values obtained using the ActiGraph algorithm (absolute percentage errors=[(observed data–ActiGraph data)÷observed data]×100). Intraclass correlation coefficients (ICC_2,1_) [24] were used to examine criterion validity between step count taken from the ActiGraph compared with those observed during the TUG and 30MWT. By convention, an ICC ≥0.75 was considered excellent, 0.60‐0.74 good, 0.40‐0.59 fair, and <0.40 poor [25].

Bland-Altman plots [26,27] were constructed to show the variability of the ActiGraph in recording step count compared with the observed data. With this technique, the mean error score and the 95% prediction intervals can be examined in graphical form. Comparisons that are in closer agreement will have a mean bias close to zero and tighter 95% prediction intervals. Statistical analyses were conducted with SPSS version 26 (IBM Corp) with the α level set at .05.

## Results

### Overview

A total of 25 older adults (n=17, 65% women) with a mean age of 79.6 (SD 8.1) years participated in experiment 1, and 30 participants (n=24, 80% women), with a mean age of 81.4 (SD 8.8) years, participated in experiment 2 (Table 1). The patients were hospitalized due to the following conditions: cardiac conditions (n=6) including hypertension, congestive heart failure, atrial fibrillation, and coronary artery disease; urinary tract infection (n=5); deconditioning (n=5); falls (n=3); acute kidney injury (n=3); exacerbation of COPD (n=2); shortness of breath (n=2); sepsis (n=2); pneumonia (n=1); syncope (n=1); leg pain (n=1); vertigo (n=1); gastroenteritis (n=1); and back pain (n=1). Table 1 describes the patients’ demographics and clinical characteristics. In general, patients were more independently mobile before admission.

**Table 1.** Demographics, comorbidities, and mobility characteristics of the patients.

Variables			Experiment 1	Experiment 2
Demographics, n			25	30
	Age (y), mean (SD)		79.6 (8.1)	81.4 (8.8)
	Sex (female), n (%)		17 (65)	24 (80)
	BMI (kg/m^2^), mean (SD)		25.6 (6.2)	25.2 (7.7)
Gait aids, n (%)			25 (100)	29 (100)
	None		12 (48)	7 (24)
	Walker rollator		12 (48)	16 (55)
	Cane		1 (4)	2 (6)
	Cane and walker		0	4 (14)
Most common comorbidities, n			30	—^c^
	Cardiac condition (hypertension, congestive heart failure, atrial fibrillation, and coronary artery disease)		25	—
	Diabetes		9	—
	Osteoarthritis		8	—
	Cancer		7	—
	COPD^b^ and Asthma		7	—
	Osteoporosis		5	—
	Kidney disease		5	—
	Early diagnosis of Alzheimer		2	—
	Parkinson		2	—
Mobility before admission, n (%)			—	30 (100)
	Independent		—	8 (27)
	Independent with cane		—	3 (10)
	Independent with walker		—	15 (50)
	1 Person assist		—	1 (3)
	1 Person assist with walker		—	2 (7)
	Independent with cane inside and walker outside		—	1 (3)
Mobility after admission			—	N=30
	Independent		—	1 (3)
	Independent with cane		—	1 (3)
	Independent with walker		—	11 (37)
	1 Person assist		—	3 (10)
	1 Person assist with walker		—	14 (47)
Mobility tests, n			23	—
	TUG^a^ (s)			
		Mean (SD)	28.5 (13.7)	—
		Median (IQR)	23.3 (7.14-61.0)	—
	Average speed (m/s)			
		Mean (SD)	0.27 (0.16)	—
		Median (IQR)	0.22 (0.09-0.84)	—
	30MWT^d^ (m-s) test 1			
		Mean (SD) (s)	—	50 (31)
		Median (IQR) (s)	—	35 (30-120)
		Percentile 25 (s)	—	32
		Percentile 75 (s)	—	67
	30MWT (m-s) retest			
		Mean (SD) (s)	—	58 (17)
		Median (IQR) (s)	—	20 (30-50)
		Percentile 25 (s)	—	50
		Percentile 75 (s)	—	70

a TUG: timed up and go.

b COPD: chronic obstructive pulmonary disease.

c Not applicable.

d 30MWT: 30-meter walking test.

### Experiment 1

The acceptability questionnaire showed that out of 25 patients, 24 patients (96%) reported no previous experience with wearables. However, 20 patients (80%) expressed their willingness to wear a wearable for a period of 5-7 days. In terms of the preferred body location, the ankle was selected by 14 patients (58%), the waist was selected by 7 patients (29%), and the thigh by 4 patients (17%). Furthermore, 1 patient did not answer this question. Among the wearable devices, the Fitbit Versa was selected by 12 patients (48%), the ActiGraph was chosen by 6 patients (24%), and the MOX1 and MMC were selected by 3 (12%) and 2 (8%) patients, respectively.

We observed that both the ActiGraph and MOX1 devices were easy to set up and enabled faster data downloads compared with Fitbit and MMC devices. Of the 4 devices, only the ActiGraph retrieved 100% of the collected data. The MATLAB function from the MOX1 retrieved 72% of the files, with 1 file containing missing data, and 7 files having a lower frequency than the specified 100 Hz setup. In addition, the MATLAB function provided by the manufacturer did not include time stamps. The algorithms on the Fitbit Versa were able to retrieve 79% of the data, with the primary issue being missing data. Similarly, the MMC algorithms retrieved 81% of the data, although some data were missing due to a time stamp error that was not identified until later in the data collection process. In addition to its high performance, the ActiGraph device also had the longest battery life and storage volume compared to all other devices. The proprietary software offered by ActiGraph allowed us to process, quickly view and extract the data using a comprehensive selection of independently developed and validated algorithms. Given the ease of data collection using the ActiGraph in the above experiment, it was selected as the most user-friendly device for further evaluation.

For the body posture assessments, the waist- and thigh-worn ActiGraph identified the lying down position correctly 73.6% and 78.2% of the time, respectively. For the standing posture, both the thigh- and ankle-worn ActiGraph achieved high identification rates of 83.8% and 82.3%, respectively. However, all devices exhibited poor performance in identifying the sitting position, ranging from 25.4% to 49.6% (Table 2).

**Table 2.** Percentage of times the ActiGraph correctly detected body posture compared with the physiotherapist recordings during experiment 1 (n=25).

Patient body posture	Experiment 1 devices’ attachment								
	Waist, %			Thigh, %		Ankle, %			
	Lie^a^	Sit	Stand	Lie	Sit	Stand	Lie	Sit	Stand
Lie	74^b^	0	0	78^b^	54	0	54^b^	0	0
Sit	11	41^b^	38	0	25^b^	15	0	50^b^	15
Stand	0	57	61^b^	1	1	84^b^	0.1	48	84^b^
Off^c^	16	1	1	21	20	1	45	3	3

a Lie: laying down.

b Percentage of time the ActiGraph device correctly identified the body postures.

c Off: percentage of time that the device detected it was off.

Table 3 displays the results of the ICC analysis, comparing the step counts during the TUG tests recorded by the ActiGraph devices worn on the waist, thigh, and ankle compared with direct observation by the physiotherapist. Data from 2 participants were excluded due to errors in registering the start and end of the tests. The ankle-worn device demonstrated the highest agreement with the physiotherapist (ICC_2,1_=0.94, 95% CI 0.85-0.97), the lowest bias (average of the mean difference=0.9 steps), and a lower percentage of error counting steps (12.8%). The waist-worn device also shows excellent agreement with direct observation (ICC_2,1_=0.85, 95% CI 0.65-0.94) but higher bias (1.4 steps) and a higher percentage of error counting steps (21%). The thigh-worn device has the lowest agreement (ICC_2,1_=0.75, 95% CI −0.21 to 0.93), the highest bias (overcounted on average 7.3 steps), and the highest percentage of error counting steps (28.8%).

**Table 3.** Intraclass correlations and percentage of error in counting steps between the physiotherapist (observer) step count and ActiGraph during the timed up and go test in experiment 1 (n=23).

Body location	Bias	ICC^a^	95% CI	*P* value	Percentage error counting steps
Waist	1.4	0.85	0.65 to 0.94	.001	21
Thigh	7.3	0.75	−0.21 to 0.93	.001	28.8
Ankle	0.9	0.94	0.85 to 0.97	.001	12.8

a ICC: intraclass correlation coefficient.

### Experiment 2

The acceptability questionnaire was completed by 25 patients. Their responses indicated that 22 (88%) had never used a wearable before, 19 (76%) would wear a device for 5 to 7 days while in hospital, and 16 (64%) were willing to wear the device daily at home, up to 3 months, as part of a research study. Furthermore, 12 (48%) patients felt motivated to move when wearing a device, and 8 (32%) would prefer to wear the device on the wrist. Intercurrences during the 24-hour protocol, include the following challenges with the devices: 1 participant had the thigh device removed and reapplied by nurses, while another participant removed both the ankle and wrist devices due to discomfort and itching. In 1 case, the wrist device was taken off because of itchiness, and another participant forgot the purpose of the devices, leading to the removal of both the wrist and thigh devices. In addition, the wrist device was loosened because of swelling, 1 participant had to remove the ankle device for 24 hours due to a major infection on the lower leg, and another was unable to wear the thigh device because the tape caused irritation.

Of the 30 patients, the ActiGraph devices were positioned on the wrist and ankle in 11 (37%) patients and on the wrist and thigh in 19 (63%) patients for the 24-hour protocol. One thigh-worn device recorded only 1:36 of data during the 24-hour protocol so the data were not included in the analysis. On average, the patients wore the device on the wrist for 22:45 (range 20:58-25:51), on the thigh for 24:36 (range 23:33-26:51), and on the ankle for 20:11 (range 8:03-26:16). Intercurrences reported while wearing the devices included itchiness at the wrist and thigh, patients removing devices due to forgetting their purpose, and patients loosening the device due to joint swelling.

The ActiGraph algorithms for the thigh-worn devices have recently changed, combining lying down and sitting as sedentary posture and including stepping detection (Table 4). The thigh-worn devices identified 100% of sedentary posture while the patient was lying down, 98% while they were sitting, and 91% while they were standing. The ankle-worn devices best identified lying (89%) and standing (84%) postures, and poorly identified the sitting posture (43.2%). In addition, the ankle-worn devices classified the position as sedentary on average 93% of the time when lying, but only 49% while the patients were seated. The wrist-worn devices performed poorly compared with the thigh- and ankle-worn devices, identifying lying down between 49% and 52% of the time and sitting and standing around 15% ‐25% of the time. The wrist-worn devices were able to identify 80% of the sedentary posture while the patient was lying down, and 71% while sitting (Table 4).

**Table 4.** Percentage of times the ActiGraph correctly detected body posture compared with the physiotherapist recordings during experiment 2 (N=30).

Patient’s body posture	Experiment 2 devices’ attachment										
	Wrist, %			Thigh, %				Ankle, %			
Sed^a^	Lie^b^	Sit	Stand	Sed	Stand	Step	Sed	Lie	Sit	Stand
Lie	84	50^c^	34	8	100^c^	0	0	92	89^c^	3	1.3
Sit	72	46	26^c^	23	98^c^	1	1	47	4	43^c^	49
Stand	76	10	66	24^c^	8	91^c^	0	13	0	13	84^c^
Off^d^	—	8	5	0	—	—	—	—	6	4	2

a Sed: sedentary (lying down + sitting).

b Lie: laying down.

c Percentage of time the ActiGraph device correctly identified the body postures.

d Off: percentage time that the device detected it was off.

e Not applicable

**Figure 2.**
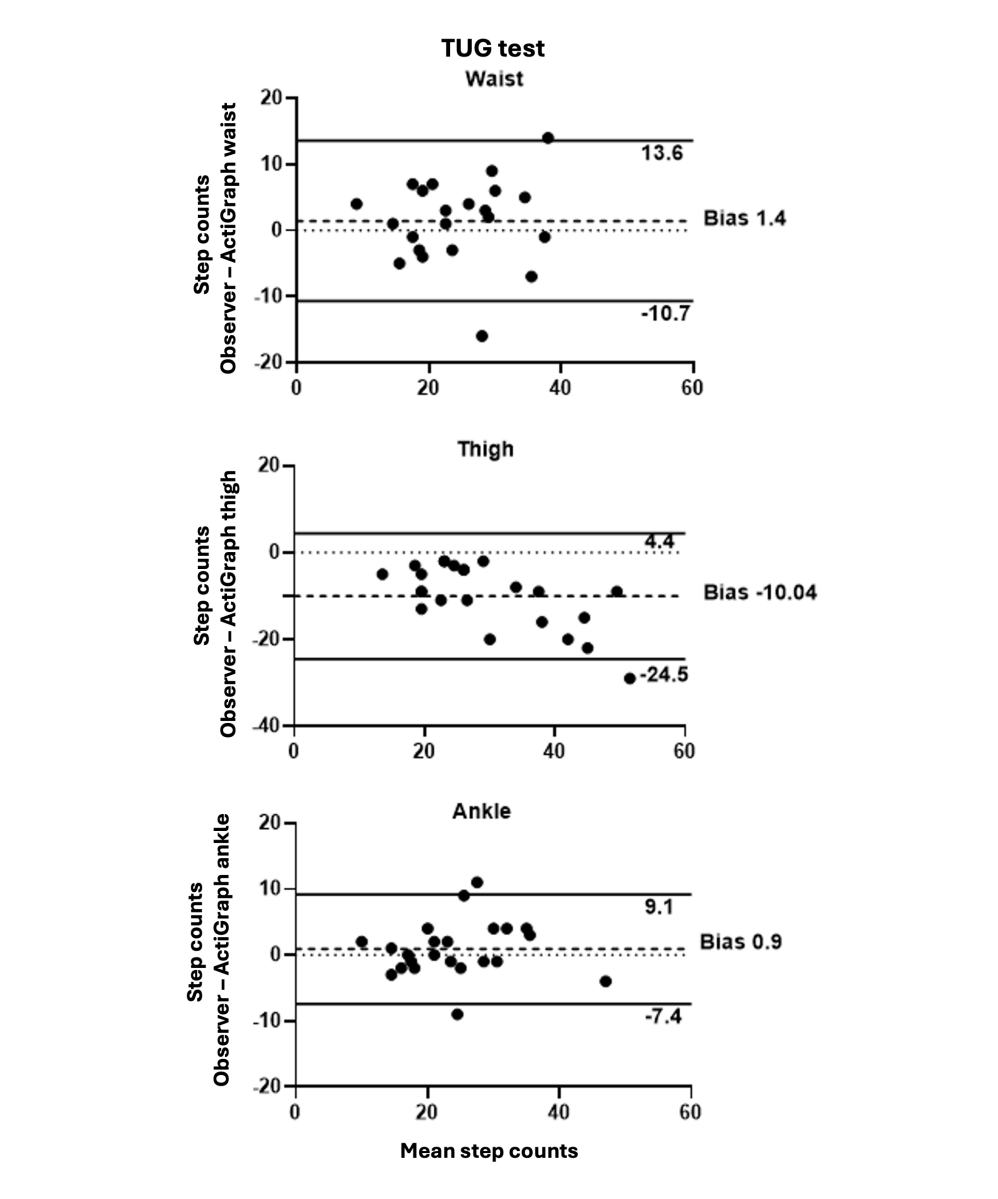
Bland-Altman plots comparing observer measurements with ActiGraph data from devices worn on the waist, thigh, and ankle during the TUG test in experiment 1. The dashed lines denote the 95% limits of agreement (SD 1.96) of the mean difference, with darker dashed lines highlighting the mean difference or bias. TUG: timed up and go.

Figure 2 shows the Bland-Altman plots of the difference between the observed step count and the ActiGraph placed on the waist, thigh, and ankle during the TUG test during experiment 1. Visual interpretation of the plots shows less bias (0.9), narrower limits of agreement (−7.4 to 9.1), and more observations closer to zero for the ankle-worn ActiGraph. In contrast, the thigh-worn device showed a larger limit of agreement, suggesting poor agreement between the 2 measurements, and a tendency to overcount steps.

During the 30MWT in experiment 2, the wrist- and thigh-worn devices showed poor ICC values and a high percentage of miscounted steps. The ankle-worn devices showed excellent reliability and on average overcounted the number of steps by 1.9% (Table 5).

**Table 5.** Intraclass correlation coefficients and absolute percentage error of steps between the physiotherapist step count and the ActiGraph during the 30-Minute Walk Test in experiment 2 (n=30).

Body location	Bias	ICC^a^	95% CI	*P* value	Percentage error counting steps
Wrist	–42.1	–0.032	–0.217 to 0.218	.62	49.4
Thigh	–36.0	0.331	–0.149 to 0.706	<.001	56.8
Ankle	1.6	0.959	0.915 to 0.981	<.001	1.9

a ICC: intraclass correlation coefficient.

**Figure 3.**
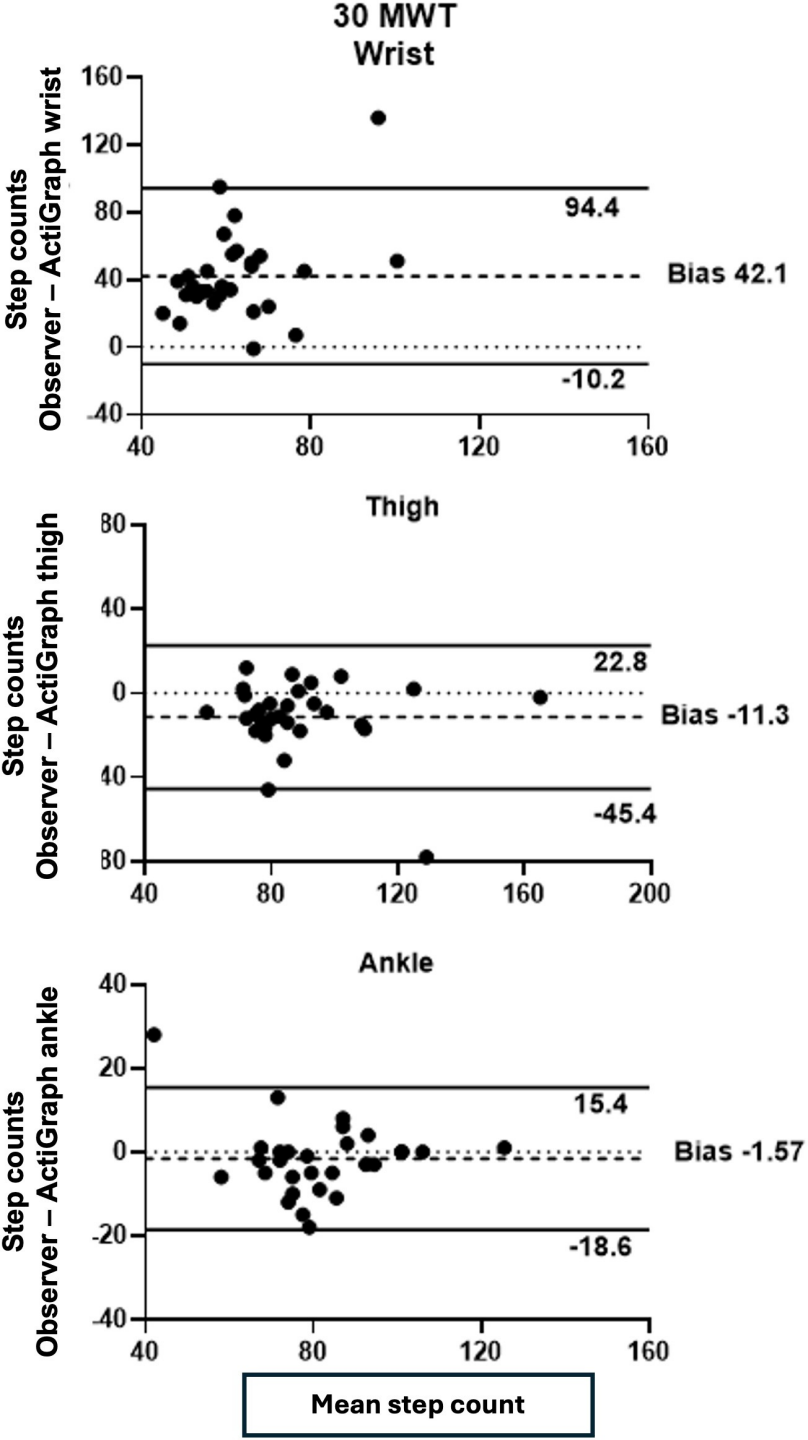
Bland-Altman plots comparing observer measurements with ActiGraph data from devices worn on the wrist, thigh, and ankle during the 30 MWT in experiment 2. The dashed lines denote the 95% limits of agreement (SD 1.96) of the mean difference), with darker dashed lines highlighting the mean difference or bias. 30 MWT: 30-meter walk test.

The Bland-Altman plots in Figure 3 illustrate the agreement between observer-rated step counts and ActiGraph step counts at the wrist, thigh, and ankle locations during the 30MWT. Among the 3 locations, the ankle-worn device demonstrated the best agreement with observer ratings, exhibiting a small bias and most observations falling within the 95% limits of agreement. In contrast, the wrist-worn device exhibited poor agreement, with the largest bias and widest limits of agreement.

## Discussion

This study aimed to identify the optimal wearable device and wear location to assess and monitor mobility among older patients during hospitalization. We observed a high level of acceptability and feasibility regarding the usability and accuracy of wearable devices for detecting and monitoring activity in older patients hospitalized for acute medical illness. Although most patients had limited experience with activity monitoring devices, they were willing to wear one while hospitalized. Patients showed a preference for the Fitbit and ActiGraph devices, worn at the ankle or waist. Both the ActiGraph and Mox1 devices were easy to set up, but only the ActiGraph allowed for the retrieval of all data collected. Overall, the ActiGraph wGT3X-BT emerged as the preferred device with superior usability, data acquisition, and management.

The ActiGraph wGT3X-BT is widely recognized as the gold standard research-grade device for wearable mobility tracking, which is consistent with the findings of this study in our population. Its previous use in hospital settings underscores its utility for monitoring physical activity and posture in clinical contexts [28-30]. For example, other studies have employed the ActiGraph to predict hospital-acquired disability [28] and demonstrated its accuracy in quantifying postures and activity levels among hospitalized adults [29]. This study supports the device’s reliability and validity among older hospitalized patients with unique challenges such as slower movement patterns and increased sedentary behavior.

However, when planning a cohort study in a hospital setting, it is crucial to consider multiple factors beyond just device accuracy, including accessibility to raw data and patient acceptability. During our study, the Fitbit Versa 1 was a popular device choice but lacked the option to download raw data directly. Instead, the download process relied on a companion device, a tablet, and any miscommunication between the 2 devices resulted in data lost. The MMC was selected for its affordability and open-source platform. However, we encountered challenges with the downloading process, which was time-consuming, and we also noted timestamp errors. The MATLAB function provided by the MOX 1 also resulted in missing data and lack of time stamps. Despite the challenges encountered with the MMC, MOX 1, and Fitbit devices in our study, it is plausible that these issues have since been addressed and resolved, underscoring the potential advancements made by these companies in their software and device functionalities. Our study highlights the importance of considering broader factors, such as data accessibility and device functionality, when evaluating the feasibility of wearable technology for hospital-based cohort studies.

Studies have suggested the lack of physical activity and immobilization during hospitalization may be more related to aspects of hospital care rather than to the patient’s diagnosis [1,31]. While performance-based mobility tests can predict functional decline and hospital discharge, they are not commonly integrated into hospital measures [32-34]. Conducting a gait speed test, for example, is time-consuming and might not be feasible on a day-to-day basis in the hospital setting. Therefore, the use of wearable technology could be an attractive option, requiring minimal time investment for both patients and health professionals. There is a substantial body of literature on the utilization of wearables in hospitalized patients [16,28,34,35]. However, the research on patient feedback regarding their experiences and perceptions of wearing the devices while hospitalized is limited [17], which is crucial for optimizing their use in health care settings. In addition, we were particularly concerned about the potential interference of these devices with patients’ medical conditions. Prolonged sitting and fluid intake, common in hospitalized patients, can lead to swelling, particularly in the ankles and wrists. We observed instances of wrist and ankle swelling in patients that resulted in the devices being removed, underscoring the importance of considering these issues when implementing wearable technology in hospital settings. Furthermore, we also considered the possibility of the devices interfering with medical equipment commonly used during hospitalization, including colostomy bags, urinary catheters, and wound dressings, as any disruption to these essential devices could compromise patient care and safety. For instance, in our first experiment, we applied an elastic band with pockets to the patient’s waist, thigh, and ankle. We noticed that the waistband was problematic because it would interfere with heart sensor wires and colostomy bags. In addition, the thigh elastic band would easily fall from the participant’s leg. Thus, in the second experiment, we disregarded the waistband and used an adhesive patch, which could also bring discomfort due to skin itchiness, as observed in our study. Considering these factors before launching a larger cohort is crucial and might save time and effort for both patients and researchers.

Our findings highlight the importance of considering both practical and contextual factors when selecting wear locations for mobility monitoring in hospital settings. While the literature supports the thigh for measuring postural behaviors and the waist or ankle for step counts, these locations may not always be feasible in clinical environments. Wrist-worn devices, although convenient and widely accepted, presented challenges such as patient discomfort due to swelling and skin irritation. This study serves as a preliminary exploration of acceptable and practical wear locations in a hospital setting, emphasizing the need to balance feasibility with the specific mobility metrics of interest.

It is well-established that sedentary behavior in hospitalized older adults is associated with a heightened risk of hospital-acquired disability (HAD) and functional decline [28,29]. Therefore, accurately measuring sedentary behavior in this population is essential for timely intervention and management strategies within the hospital setting. Body posture poses a challenge in accurate measurement, ideally requiring the use of at least 2 devices. For instance, thigh-worn devices have been reported as optimal for placing accelerometers to determine sedentary behavior (ie, lying and sitting). In this regard, the thigh-worn inclinometer algorithm provided by ActiGraph uses a movement threshold and thigh angular orientation to distinguish lying and sitting from standing and stepping [36]. Compared with other algorithms such as the activPAL, the ActiGraph’s thigh angular parameter improves the classification of sitting posture, even when the participant has their legs crossed or stretched, in both laboratory and free-living conditions [22,37]. Waist-worn devices are effective for differentiating lying from sitting but not from sitting and standing, while ankle-worn devices distinguish lying and standing. In our study, we tested the ActiGraph on the waist, thigh, and ankle, and in experiment 1, all wear locations performed well for lying and standing but poorly for sitting. We believe that the thigh elastic band likely changed position when participants transitioned from lying to sitting, changing the orientation of the ActiGraph, thus affecting the algorithms’ ability to detect sitting posture. In experiment 2, we incorporated a wrist-worn device due to its user-friendly nature. To mitigate the issue encountered in experiment 1 with the elastic band, we adopted adhesive patches to secure the devices on the thigh and ankle. The thigh device exhibited the highest performance across various postures, followed by the ankle device, which showed adequate accuracy in detecting lying and standing postures. However, the wrist device, despite its ease of use, performed poorly in detecting all body postures. Our findings are supported by the literature [17,29], and we recommend using an accelerometer on the thigh and ankle to capture more detailed information on sedentary behavior.

Our investigation on step counts showed that the ankle is the most accurate body position to capture this metric. This finding aligns with previous research examining the validity of ankle-worn ActiGraph devices in conjunction with the lower frequency extension filter for step counting among hospitalized older adults [16,38]. In their study, Webber and St John [38] demonstrated that the ActiGraph positioned on the ankle was comparable with direct observation (ICC=0.94, median absolute error=2.5%) for monitoring step counts during the 10-meter walk test in hospitalized older adults. In addition, Anderson et al [16] reported that the ActiGraph positioned on the ankle (mean difference=−0.85 steps, ICC=0.99) accurately records step counts in hospitalized adults during free self-selected walking. By leveraging the ankle and thigh as the placement site for capturing mobility, researchers and health care practitioners can enhance the accuracy of monitoring and promote more effective interventions aimed at improving mobility and overall health outcomes in hospitalized adults and similar populations.

A potential limitation of this study is the bias introduced by patients wearing multiple devices simultaneously in experiment 1. The discomfort or inconvenience of wearing several devices may have influenced their feedback, as they were likely focused on the overall experience rather than evaluating each device individually. However, the primary goal of experiment 1 was to assess the feasibility of using multiple devices in a hospital setting, with a focus on understanding the practical and real-world challenges of device wearability, data retrieval, and integration in a clinical environment. While this may introduce some bias in the acceptability results, it does not diminish the value of these insights into how devices function together in practice.

Building on these findings, our study has several strengths, including identifying optimal devices and placement sites for wearable data collection, as well as validation through comparison with a gold-standard observer. However, it is important to note that our free-living protocol in experiment 2 was limited to a 24-hour duration. Despite this constraint, we were able to gather valuable insights, particularly regarding the feasibility and integration of wearable technology in hospital settings.

In conclusion, our study found that the ActiGraph wGT3X-BT was the most feasible device for assessing and monitoring mobility among older hospitalized patients. The ActiGraph’s thigh-worn algorithm accurately detects sedentary behavior under supervised conditions and, when paired with a device at the ankle, provides detailed information on lying and sitting postures. In addition, our findings indicate that step counts can be accurately detected using the low-frequency extension with devices on the ankle. Therefore, we recommend the use of two devices, at the thigh and ankle, to accurately measure sedentary behavior and step count among older people in a hospital setting. Longer-term studies are warranted to evaluate the use of wearable data for predicting health outcomes after hospitalization and for informing clinical decision-making and efforts to promote early mobility among older hospitalized patients.

## Abbreviations

30MWT: 30-meter walking test
ICC: intraclass correlation coefficient
MMC: MetaMotionC
TUG: timed up and go
